# 3-D Surface Morphological Characterization of CAD/CAM Milled Dental Zirconia: An In Vitro Study of the Effect of Post-Fabrication Processes

**DOI:** 10.3390/ma15134685

**Published:** 2022-07-04

**Authors:** Ashwini Patil, Davidson Jebaseelan

**Affiliations:** School of Mechanical Engineering (SMEC), Vellore Institute of Technology, Chennai 600127, India; p.ashwinivit@gmail.com

**Keywords:** CAD/CAM machine, sandblasting, polishing, alumina particles, 3-D surface roughness

## Abstract

Objective: To investigate the effect on zirconia surface of the post-fabrication surface treatments on the morphological characteristics and mechanical properties of CAD/CAM milled dental zirconia specimens as well as to identify the critical parameters in the measurement of oral retention under in vitro circumstances. Method: The zirconia specimens (N = 20, *n* = 4) were subjected to CAD/CAM milling and divided into five groups. The specifications were: Group G1—sintered; Group G2—sintered followed by a polishing process; Group G3—sintered followed by polishing and sandblasting with alumina particles Al_2_O_3_ (110 µm); Group G4—sintered followed by sandblasting; Group G5—sintered followed by sandblasting with polishing as the end process. All the groups were subjected to Fretting wear tests, 3-D surface roughness measurements, and Vickers’s Micro hardness tests. Investigation of the phase transformation using XRD, and surface feature examination using SEM were also carried out. Additionally, one-way ANOVA, Tukey, and Pearson correlations were statistically analysed. Results: The fabrication processes had a significant effect on the performance of zirconia specimens in all the groups (*p* > 0.05). Specimens that underwent polishing as the last process exhibited lower surface roughness. The monoclinic phase of zirconia was observed in all the specimens before and after wear except for those in the G2 and G5 groups, where polishing was the end process. In G5, the post-wear surface properties revealed lower surface roughness and hardness. Further, the SEM and 3-D topography show grooves as seen by the dale void volume (Vvv) values; shallow valley depth (Svk); micro craters; and wear track. Conclusion: Specimens in G5 that were subjected to multistep post-fabrication process, namely sandblasting followed by polishing, yielded better results when compared to those in the other groups (G1, G2, G3, and G4). G5 with an interlayer of alumina is recommended for clinical applications due to its enhanced surface properties, mechanical properties, and low wear.

## 1. Introduction

CAD/CAM (Computer-Aided Design/ Computer-Aided Manufacturing) milling of a pre-sintered blank in a full-contour form is often done in monolithic zirconia restorations in clinical practice. Due to their optical, mechanical, and biological properties, the Yttria-stabilized Tetragonal Zirconia Polycrystals (Y-TZP) are increasingly used for high-strength ceramic crown bridges and abutments [1,2,3,4]. This is significant in terms of clinical practice because polished monolithic zirconia has the potential to be a versatile restorative material. To be a multipurpose restorative substance, this material outperforms all-ceramic restorations in terms of mechanical qualities and reduces the risk of porcelain chipping. Furthermore, when compared to an all-ceramic crown, less tooth structure reduction is required, resulting in a more natural tooth structure [5]. Prosthetic dentistry using Yttria-stabilized zirconia crowns is done to restore missing and damaged teeth due to their aesthetic and mechanical properties. The dentist’s chairside clinical correction methods are largely required to achieve appropriate occlusal contact. The zirconia surface characteristics can also be affected by the milling process [6].

The post-processing treatments such as grinding, polishing, glazing, and sintering on Y-TZP CAD/CAM milled surfaces influence their surface microstructure and roughness [7,8]. Additionally, the sequence of the post-processing, say, polishing after grinding, is observed to either improve or degrade the mechanical properties of Y-TZP [8,9,10,11]. Previous reports indicate that there is a strong correlation between the flexural strength and the surface roughness of Y-TZP due to grinding and polishing. Another study concluded that grinding affected the Y-TZP surface characteristics, increasing their fatigue strength due to the phase transformation mechanism, while polishing as the end procedure decreased the surface roughness but had little effect on the fatigue strength [8].

Clinical adjustments such as grinding are usually performed to improve the emergency profile and occlusal relations. Due to surface defects that cause phase transformations, manufacturers usually recommend a polishing protocol after grinding [9,11,12]. Sandblasting with 250 µm alumina particles as the final procedure provided the highest surface roughness, according to Alao A.R et al. [13]. In their research, the polishing followed by sintering produced the lowest Ra value. De Souza et al. [10] observed a surface roughness of 0.32 µm in the as-sintered condition. When subjected to grinding alone, the surface roughness value was 0.87 µm, and when subjected to grinding with polishing, the roughness was reduced to 0.55 µm. This study revealed changes in fracture resistance and fatigue strength due to changes in surface roughness. This results in a tetragonal to monoclinic grain transformation, which starts from the surface and is seen to grow as micro-cracks into their sub-surfaces. As a result, the material’s sensitivity to low-temperature deterioration is exacerbated by seepage of moisture and fluids. The surface energy of zirconia is not particularly high. Zirconia does not have chemical adhesive potential or etchability [14]. To develop a long-lasting relationship, more research into mechanical interlocking is required. However, there have been few studies on glazing and stain wear [15,16], and zirconia is the material that loses these extra layers the most. Therefore, more research is needed.

Several studies have been undertaken to lessen the damage caused by zirconia on natural teeth restorations by a range of finishing processes that include polishing, fine-grinding, coarse-grinding, glazing, reglazing, and heat treatments [17,18,19,20]. Post-processing techniques after adjustments restores the surface smoothness so that they do not impede the mechanical performance and the wear of the teeth/monolithic restoration. Nevertheless, there is a need to study the peaks and valleys on the surfaces left by the polishing traces, which could increase oral retention in valleys created by polishing bur.

The results from several studies performed under different wear conditions and with differences in the geometry of the natural teeth were of contradictory nature. Among the four common finishing procedures, polishing as an end process has shown promising results [21,22,23]. These results have made the dental community adopt polishing as the end process. However, as clinical adjustments are made while polishing, detailed morphological studies are warranted.

For assessing wear, a majority of the authors favour the analysis of vertical linear loss. Few studies have analysed the total loss of volume due to the wear process. According to DeLong et al. [24], the wear loss can be better quantified by comparing successive 3-D pictures because volumetric loss is linear with time, whereas depth or area measures are highly dependent on occlusal variables. Additionally, the quantitative analysis using 3-D profilometry facilitates the process of relating changes in the enamel texture to active wear mechanisms. In most of the literature reports [7,8,9,11,13,21,23,25], simple profile surface measures like Ra and Rq are used to address the overall surface quality, whereas areal parameters have proved to be more useful. Sidambe et al. [26] showed in their studies that 3-D surface parameters, Sa and Sq, and the topography have better readability than 2-D surface parameters Ra and Rq.

Given this background, the present research work investigates the effect of the surface treatments on the morphological characteristics and mechanical properties of Y-TZP. An effort is made to study the friction and wear behaviour of Y-TZP under reciprocating sliding in the presence of artificial saliva. Examination of the results of the morphological studies of the surfaces will be helpful in providing direction to dentists in chairside therapeutic modifications. The hypothesis of this study is that there are significant differences in the wear behaviours of the Y-TZP plate tribo-pairs subjected to different surface treatments.

## 2. Material and Methods

**Figure 1:**
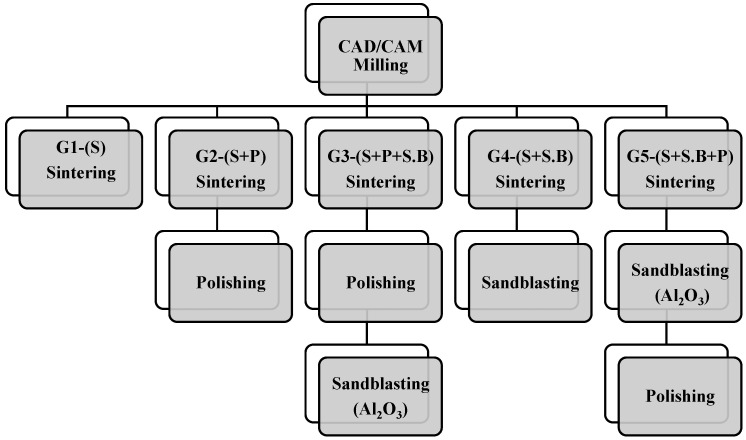
Categorisation of the Y-TZP specimens based on the different post-fabrication processes.

The study used Y-TZP pre-sintered discs (IS-TR ZD9810, Jyoti ceramic industries Pvt. Ltd., India). The pre-sintered discs were milled using a dental CAD/CAM machine (Ceramill Mikro, Amann Girrbach, Koblach, Austria). The milling process was carried out in dry conditions. The samples were subsequently milled to a dimension of 10 mm × 10 mm × 5 mm. Later, the samples were subjected to sintering with a heating and cooling rate of 20 °C/min, a holding rate of 2 h, and a temperature of 1450 °C. The samples were polished using an abrasive mounted stone, mister’s cone, and a diamond polisher. They were subsequently sandblasted with Aluminium oxide (Al_2_O_3_) particles of size 110 µm at 10 mm distance for 15 s at a pressure of 2 bar. Figure 1 represents the categorization of the Y-TZP specimens based on the different post-fabrication processes. The specimens were categorized into five groups, namely G1, G2, G3, G4, and G5. The specifications are: Group G1—sintered; Group G2—sintered followed by a polishing process; Group G3—sintered followed by polishing and sandblasting with alumina particles; Group G4—sintered followed by sandblasting; Group G5—sintered followed by sandblasting with polishing as the end process.

### 2.1. Sample Characterisation

A scanning electron microscope (SEM) (XL3 FEG, FEI, Eindhoven, The Netherlands), employing a high vacuum, 5 kV voltage, 60 µA current intensity source was used to examine the surface modification of the specimens subjected to different surface treatments. XRD (Rigaku, Japan) was used to obtain the X-ray diffraction pattern of the specimens. The measurements were carried out using Cu K-α radiation with a wavelength of 0.15406 nm in the 2θ range of 10–80° with a scanning step of 0.02°. The 3-D surface roughness of the material was determined using a non-contact surface roughness tester (Talysurf CCLITE, Magnification of 20×). Five readings were randomly taken on the surface of specimens in each group. The 3-D parameters, which include surface roughness (arithmetic average) (Sa), root mean square (Sq), dale void volume (Vvv), and reduced valley depth (Svk) were analysed. The Vickers’s Microhardness test (HMV-G series, Shimadzu, Kyoto, Japan) was performed with five indentations per sample under a load of 1 kg for a duration of 15 s.

### 2.2. Tribology Test Parameters

The wear study was carried out on the Y-TZP specimens with a ZrO_2_ ball as the antagonist mounted on the reciprocating ball-on-plate (Fretting machine, Ducom, India) tribometer. Four specimens were used from each group. The diameter of the antagonist zirconia ball was 6 mm, while the dimensions of the zirconia block were 10 mm × 10 mm × 5 mm. The wear test parameters were evaluated at a temperature of 37 ± 1° under a constant applied load of 10 N with a stroke length of 0.5 mm and a frequency of 5 Hz for a duration of 1 h [22,27,28,29]. The artificial saliva as a medium was prepared with 1000 mL of distilled water. The chemical composition of artificial saliva was carried out in accordance with the literature [30]. The frictional force was recorded during the test, and the friction coefficient was subsequently determined.

### 2.3. Data Analysis

Statistical analysis was conducted using Minitab version 19 software for Windows (Minitab Inc., State College, PA, USA) with a confidence interval of 95%. The Shapiro-Wilk test was used for normality test of data, ANOVA was performed for different fabrication processes and the results were compared to obtain the mean of Sa. All multiple pairwise comparisons were performed using the Tukey test. The Pearson correlation was used to determine the association between the 3-D parameters (Sa, Svk, Vvv), the Coefficient of Friction (COF), and the hardness before and after wear.

## 3. Results

### 3.1. SEM Characterisation and 3-D Profilometry

**Figure 2:**
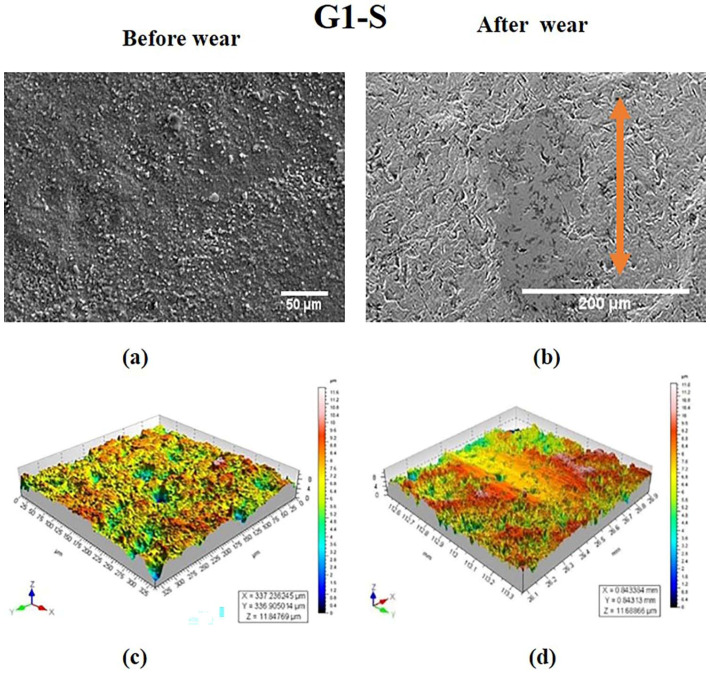
SEM images of specimen in G1 group at magnifications of (**a**) 50 μm and (**b**) 200 μm. 3-D surface morphology profile (**c**) before wear (**d**) after wear. Wear track is indicated by arrow.

The 3-D surface profiles of specimen in group G1 before and after wear are represented in Figure 2c,d. The corresponding SEM images are shown in Figure 2a,b. The sintered group G1 was characterized by surface irregularities as seen in Figure 2a,c. After wear, the wear track revealed a smooth surface (Figure 2b,d). Hence, it can be concluded that the wear of the material was minuscule.

**Figure 3:**
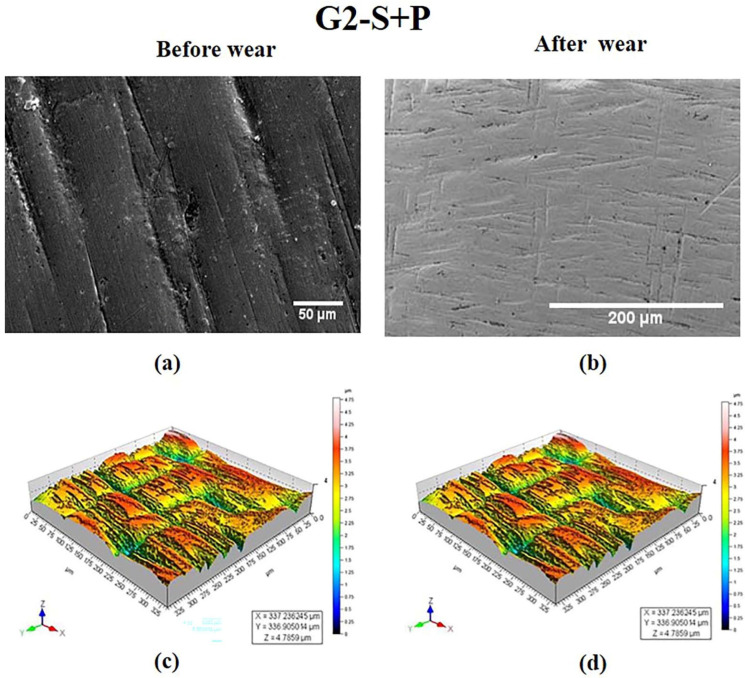
SEM images of specimen in G2 group at magnifications of (**a**) 50 μm and (**b**) 200 μm. 3-D surface morphology profile (**c**) before wear (**d**) after wear.

For the specimen in the G2 group, the SEM and 3-D roughness contour (Figure 3a,c) revealed polishing traces with deep grooves and pits. The 3-D surface characteristic image in Figure 3c revealed high peaks and valleys with micro irregularities all over the surface induced by the polishing process. After wear in the G2 group, no noticeable wear track was observed in the 3-D topography image (Figure 3d) since the surface was covered with polishing traces. Though the SEM image in Figure 3b did not reveal wear track, surface undulations could be observed.

**Figure 4:**
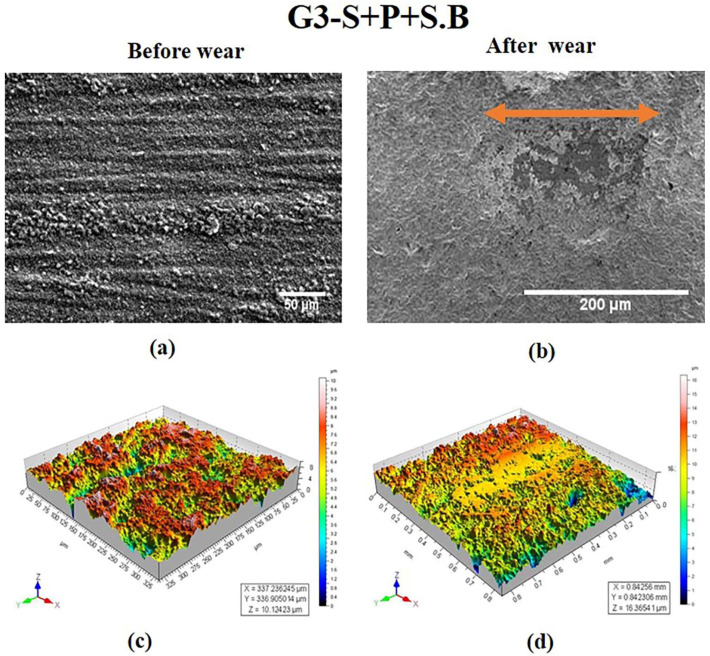
SEM images of specimen in G3 group at magnifications of (**a**) 50 μm and (**b**) 200 μm. 3-D surface morphology profile (**c**) before wear (**d**) after wear. Wear track is indicated by arrow.

From the SEM image (Figure 4a) of the specimen in the G3 group, polishing traces with shallow depth were observed. This is further evident from the 3-D image in Figure 4c. The process of sandblasting filled the grooves and pits. Additionally, the large debris was closed by the sandblasting particles leaving very small polishing traces and delamination. The 3-D topography (Figure 4d) after wear in the G3 group specimen revealed a wear track.

**Figure 5:**
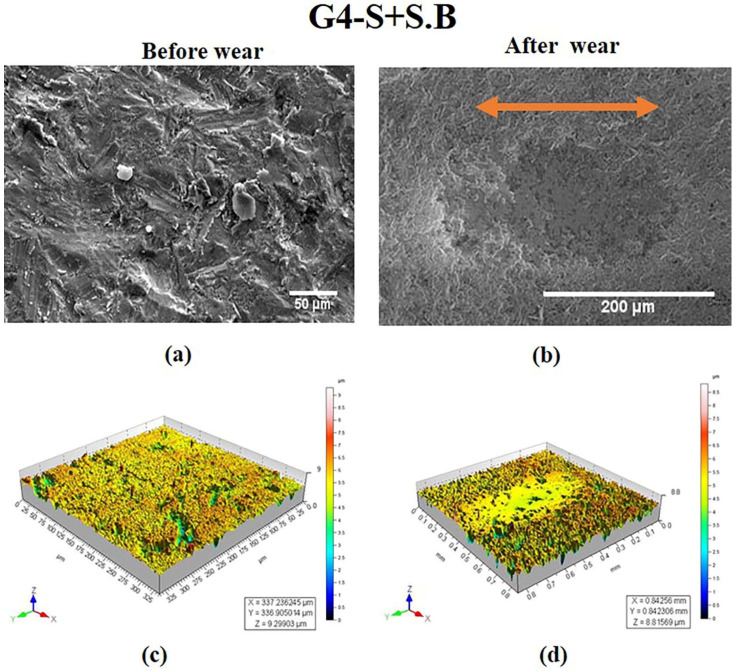
SEM images of specimen in G4 group at magnifications of (**a**) 50 μm and (**b**) 50 μm. 3-D surface morphology profile (**c**) before wear (**d**) after wear. Wear track is indicated by arrow.

The specimen in the G4 group was subjected to sintering followed by sandblasting, which was responsible for random orientations on the surface as evident from Figure 5a. The sandblasting particles adhered to the base material, while smaller particles were found on the surface. Figure 5c shows a textured surface due to sandblasting instead of a peak or a valley. After wear, the SEM image as well as the 3-D topography image displayed wear track due to the removal of alumina particles (Figure 5b,d).

**Figure 6:**
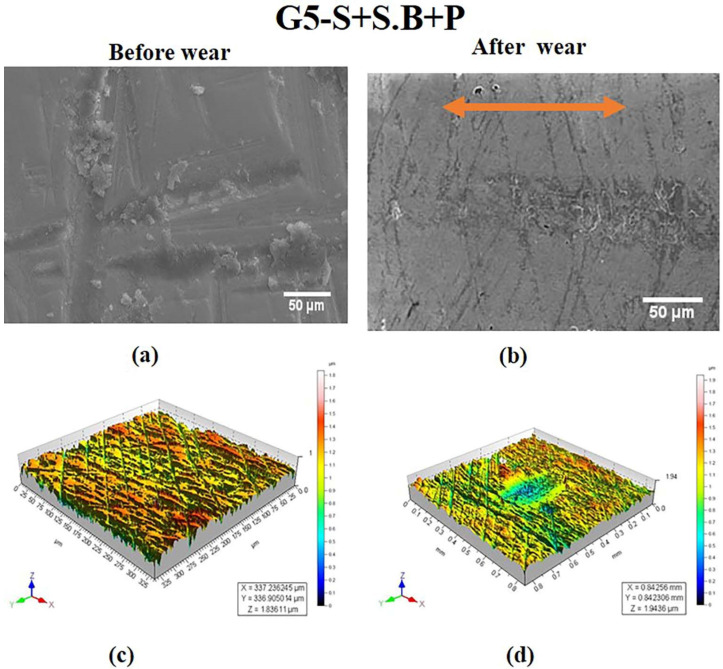
SEM images of specimen in G5 group at magnifications of 50 μm (**a**) before wear and (**b**) after wear. 3-D surface morphology profile (**c**) before wear (**d**) after wear. Wear track is indicated by arrow.

In the case of the specimen in the G5 group, polishing traces similar to the ones seen in G2 (Figure 3a) were observed. Observation of the microstructure in Figure 6a revealed micro craters with deep sharp scratches on the surface. The absence of pitting is attributed to the fact that sandblasting performed as a second stage technique caused the particles to seal all the pits. Several peaks and valleys were observed in the 3-D profile in Figure 6c. In spite of polishing traces, the 3-D topography of the specimen after wear revealed the wear mechanism as seen in Figure 6d. The peaks were removed due to the adherence of alumina particles to the surface. The depth of the wear was at a surface level as observed in the SEM image (Figure 6b).

Table 1 presents the surface roughness values of the specimens in different groups, both before and after wear. The values from Table 1 indicate a decline in the surface roughness of the material after wear in all the groups. The specimen in the G5 group exhibited the lowest average surface roughness (Sa = 0.182 ± 0.018 µm), indicating the least resistance to wear. The grooves produced after wear were minimal, indicated by the values of Vvv (0.032 (0.003) µm³/µm²) and Svk (0.312 (0.035) µm). Hence, G5 possessed a shallow depth. The polishing process is the final procedure in both the G2 and G5 groups. The specimen in the G5 group exhibited the lowest 3-D values due to sandblasting using alumina being the intermediate process, hence the resistance to polishing bur to impinge the material deeply.

S Lou et al. [31] made attempt to study the 3-D surface parameter (Sa, Sq, Ssk, Svk, Sk) to better understand the surface topography of 3-D parts. The 3-D topographies shown in Figure 2d, Figure 4d, Figure 5d and Figure 6d revealed wear track in the G1, G3, G4, and G5 specimens. In G2, the wear track was non-traceable as a result of the groove produced due to polishing. The highest surface roughness of 1.049 ± 0.105 µm after wear was observed in the G3 group. In the present study, the 3-D parameters were statically analysed and were found to have strong positive correlation (Svk-Vvv (r = 0.999), Sa-Svk (r = 0.956), and Sa-Vvv (r = 0.965)) as observed in the statistical tests.

### 3.2. X-ray Diffraction

**Figure 7:**
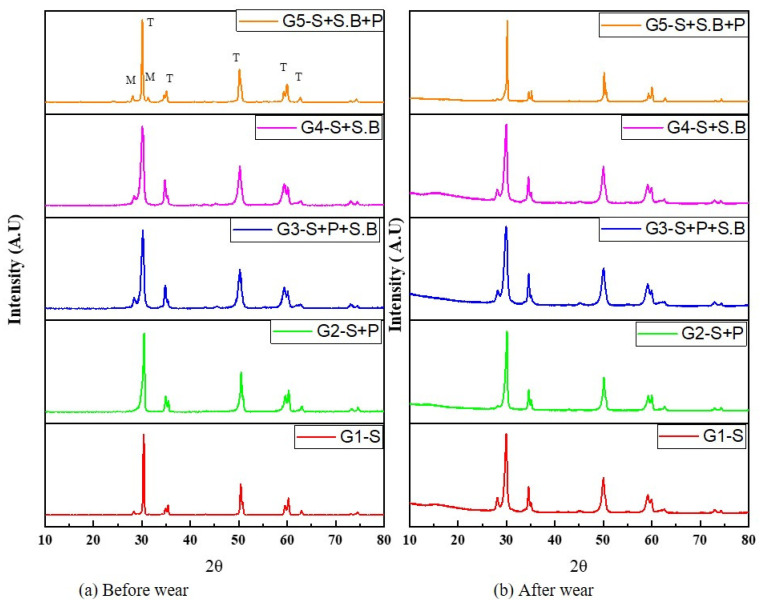
X-ray diffraction patterns of Y-TZP specimens (**a**) before and (**b**) after wear.

The X-ray diffraction patterns for specimens from various groups are shown in Figure 7. By comparing against the JCPDS database [32], the crystalline phases were identified. All of the zirconia specimens had a monoclinic peak with an orientation of m (−111). Minor peaks were found at 28.2° and 31.4°, respectively. The material’s X-ray diffraction pattern revealed that it was mostly made up of the tetragonal zirconia crystalline phase. The major peaks of tetragonal ZrO_2_ were identified at 2θ values of 30.21, 34.91, 50.41, 60.21, 63.51, and 74.21, which correspond to the (111), (200), (220), (311), (222), and (400) diffraction planes of the tetragonal Y-TZP crystal, respectively. These findings are consistent with those found in the investigation by Alao A.R. et.al. [13]. After wear, the investigation discovered a diffraction peak shift with broadening on the t phase. Except in the G5 group, wear testing had no discernible effect on the phase transformation rate, regardless of the surface treatment used.

### 3.3. Hardness Measurements

**Figure 8:**
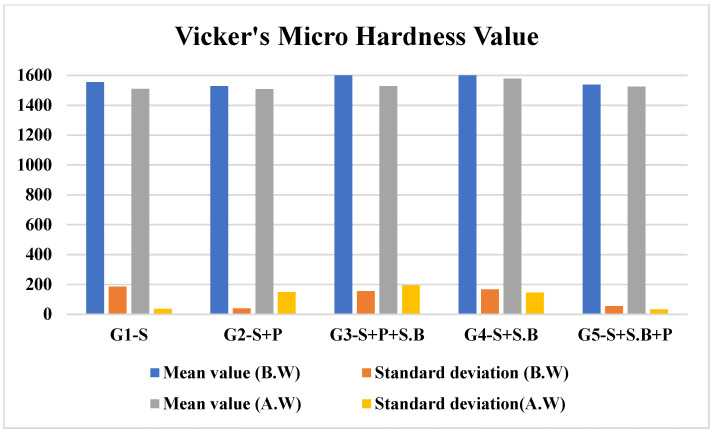
Vickers’s microhardness values for specimens in different groups before and after wear.

Microhardness testing is a method of determining a material’s hardness or resistance to deformation when the test samples are not suitable for macrohardness. The hardness values of the various groups are presented in Figure 8. It is observed that, before wear, the hardness values varied significantly with the post-processing treatments. Specimens that were subjected to sandblasting as the end process (G3 & G4) reported higher hardness values. However, the hardness after wear was found to decrease. This is attributed to wear and saliva, which functioned as a medium in the fretting wear mechanism. The hardness values of G2 (1508 ± 149.57) and G5 (1524 ± 33.77) were the least among all the groups after wear. Hence, it can be inferred that the specimens in G2 and G5 exhibited better resistance to those in the other groups. The hardness values obtained in the present study are in agreement with the values reported in the literature.

### 3.4. Analysis of Variance

ANOVA, which stands for Analysis of Variance, is a statistical test used to analyse the difference between the means of more than two groups. Table 2 reveals the result of the one-way ANOVA to determine the influence of different processes on the arithmetic mean roughness Sa. The values indicate that Sa was significantly influenced by different fabrication processes (ANOVA, *p* > 0.05). The normality of the variance of all used data was confirmed by the Shapiro-Wilk test. Tukey’s method considers all possible pairwise differences of means at the same time. The Tukey HSD tests demonstrated that the differences between all the groups were statistically significant (all *p* > 0.05).

**Figure 9:**
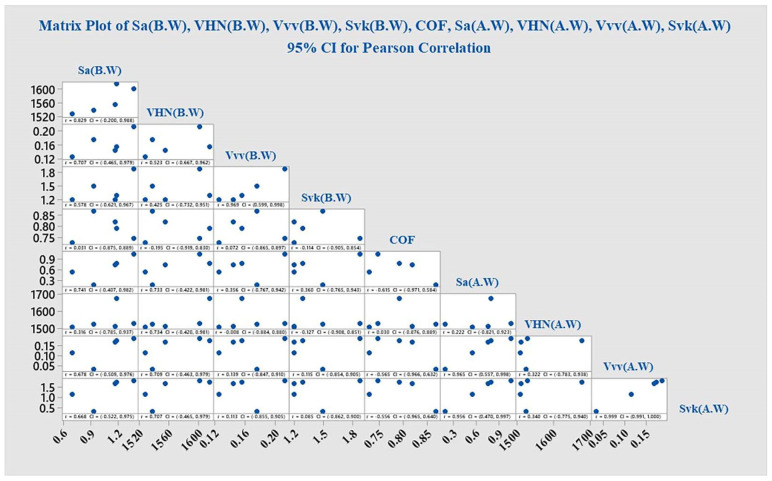
Pearson correlation between the 3-D parameters (Sa, Svk, Vvv), hardness (HVN), and COF, before and after wear.

Pearson Correlation Coefficient is a type of correlation coefficient which represents the relationship between the two variables. Figure 9 Pearson correlation between the 3-D parameters (Sa, Svk, Vvv), hardness (HVN), and COF, before and after wear. In the present study, it was observed that values of Sa (A.W), Vvv (A.W), and Svk (A.W) have a positive correlation. Thus, it can be concluded that the wear mechanism has effect on the valley and depth of the material.

### 3.5. Coefficient of Friction (COF)

**Figure 10:**
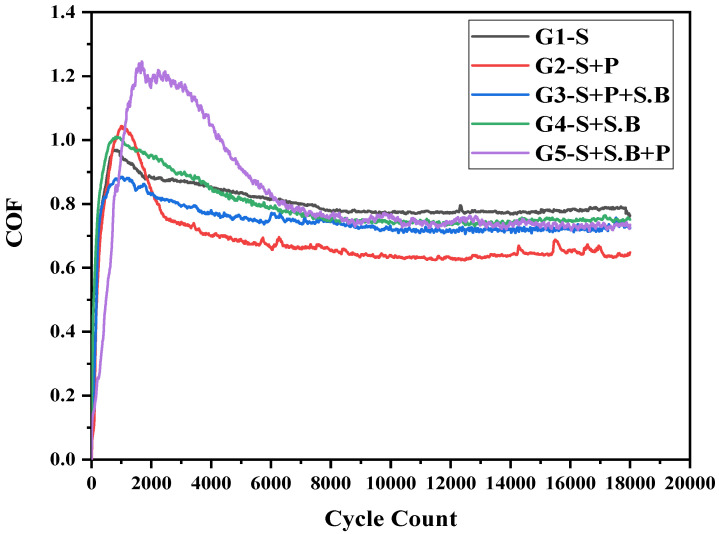
Coefficient of friction as a function of the cycle count for Y-TZP specimens in different groups.

A proper of understanding of friction and the ways to manage it are of paramount importance for a successful dental practitioner. A surface is classified as frictional if it resists the relative motion between itself and another surface in contact. The coefficient of friction (COF) is an important parameter used to identify intrinsic tribological characteristics. In this work, the coefficients of friction between the zirconia ball and different flat square specimens were determined. Typical variations in the friction coefficient, as a function of the number of cycles, were examined at 10 N load under the artificial conditions for about 1 h. The highly polished zirconia occlusal surface can result in a much-reduced antagonist enamel wear [33]. The saturation value of the coefficient of friction was found to decrease. Additionally, the COF value of zirconia attained a steady-state regime between 0 and 0.819, after a running-in short period as depicted in Figure 10. The results are in agreement with literature reports [21]. The COF for G2 was 0.728, which was the least among all the groups. The surface roughness and hardness of the G2 specimen were 0.507 ± 0.034 µm and 1508 ± 149.57, respectively. After wear, the hardness and surface roughness showed a decline in mechanical properties as evident from SEM and 3-D topography images. The images also revealed local plastic deformation of the material. Hence, it was concluded that the wear has a marked influence on the material for the given parametrical conditions. Practical applications require that the wear of the material should not initiate micro-cracks on the material, which might lead to its propagation and the transformation of the material into the monoclinic phase. Since the material is ceramic, the crown may fail catastrophically, which is undesirable in dental applications.

## 4. Discussion

Three-dimensional topographic studies were carried out to investigate the surface characteristics of the ceramic (zirconia) specimens using both qualitative and quantitative measurements (Sa, Svk, Vvv). The results of the measurements are presented in Figure 2, Figure 3, Figure 4, Figure 5 and Figure 6 and in Table 1. It was observed that the surface roughness of the specimens varied among different specimen groups, due to the post-sintering processes such as sandblasting and polishing. The 3-D optical profilometer was employed in our investigation to obtain more detailed 3-D data for standardized surface area via non-contact scanning. The 3-D optical profilometer gives detailed insights into the surface morphology of the specimens subjected to various post-processing treatments. The present research revealed that the surface roughness, such as pores, scratches, and texture, could be essential in the tribological evaluation due to the antagonist interaction with the counter face. The wear behaviour of the polished zirconia showed the least wear rate among all the groups. The 3-D morphology and SEM micrographs revealed polishing burr marks and scratches. However, no wear track was noticed in the G2 group.

Replicating the clinical process, this study investigated the zirconia surface after sandblasting and polishing. The SEM analysis showed morphological differences between the five specimen groups. While shallow scratches were visible in the G5 specimen, deep grooves were observed in the G2 specimen. The G1 specimen showed irregular surface morphology with shallow scratches, fine irregularities, and pores across the surface.

Sandblasting is one of the dental procedures which increases the surface roughness and makes the surface texture irregular. Sandblasting and its impact were studied by several authors. Jenni Hjerppe et al. [34] studied different surface treatment effects on the flexural strength, bending strength, and the surface roughness of zirconia. Chintapalli et al. [35] discovered defects, deep sharp scratches, grain pull-out, and micro craters in materials sandblasted with 110 µm and 250 µm sized alumina particles. This can be explained by the high energy impact on the material as well as the effect of airborne particle abrasion, which is dependent on the particle size, particle shape, abrasion distance, and pressure.

The process of sandblasting caused significant changes in the surface roughness values (G3 = 1.049(0.105) µm), (G4 = 0.794(0.052) µm) of the zirconia specimens. However, the surface roughness significantly reduced after polishing as seen by the roughness values of the G2 and G5 specimens (G2 = 0.507 (0.034) µm, G5 = 0.182 (0.018) µm). Earlier, Himaidouch et al. [36] had suggested a three-way procedure for grinding which would be sufficient and may enable the dentist to abstain from subsequent polishing procedures. However, in the present study, it was observed that sandblasting followed by polishing (G5 specimen) significantly reduced the roughness by removing the loosely attached surface grains and by avoiding their direct impingement on the substrate. The sandblasting also eliminated the pits and the less deep polishing burrs on the surface (Figure 6a,c and Table 1). Thus, significant differences were noticed in the surface roughness of zirconia specimens subjected to different post-fabrication processes, thus confirming the hypothesis of this study.

The most noteworthy observation of the present study is that Sa-Svk, Svk-Vvv, and Sa-Vvv have a substantial correlation. The G5 group possessed shallow oral retention. There were no studies identified by the author which investigated the surface roughness (Sa), Dale void Volume (Vvv), and reduced valley depth (Svk) before and after wear tests. Therefore, no further comparison needs to be made with the results reported in earlier studies.

Polishing resulted in delamination and grooves on the zirconia surface, which was observed (Figure 3) in the SEM image and 3-D topography. In this study, it was found that the polishing process reduced the surface roughness (G2= 0.507 (0.034) µm). However, subsequent interlayers of alumina oxide, as a result of the sandblasting process, further reduced the surface roughness as seen in the G5 specimen (G5 = 0.182 (0.018) µm).

Ceramic restoration generally requires occlusal and contour adjustment, which results in a rough surface that leads to gingival inflammation, plaque accumulation, wear of the opposing teeth, and reduction in ceramic strength. Smooth surface is tried by reglazing, which is time-consuming and inconvenient [37,38]. As an alternative, the chairside finishing and polishing procedures are commonly recommended [39].

It is reported in literature that the glazing procedure produces good surface smoothness [40]. It can thus be concluded that ceramic restorations are benefitted from a polishing procedure after adjustment. However, the matter remains contentious due to different measuring parameters and material and polishing system combinations employed in prior investigations. While surface roughness is reported to be affected by milling and post-milling procedures [41], the present study reveals that the polishing procedure is found to have a significant role in reducing the surface roughness among the specimens in various groups. This was evident from the 3-D parameters, SEM analysis, and 3-D topography, which revealed deep grooves and pits on the surface.

The general consensus among dentists is that the roughened ceramic surface should be polished for better aesthetics, longer lifetime, and to prevent or reduce quick wear of opposing teeth [21,22] by adopting surface grinding procedures. Furthermore, smooth surfaces reduce plaque accumulation and retention of bacteria [38].

The results showed that roughness was strongly dependent on material and fabrication, which is consistent with earlier research [12,18,42,43,44]. The polishability of dental ceramic is normally tested in vitro on flat specimens for various durations and rotational speeds. During the polishing technique, the press-on force is typically not controlled, and this issue is not even discussed in most research studies.

High pressure of the two surface interactions and articulation can result in enhanced stress concentration. As a result of the asperity, further damage and cracking may occur. The uneven edges result in stress concentration leading to crack development, fatigue, and third-body wear.

The rough surface can increase the COF and may result in unwanted damage of the antagonist [33]. Furthermore, the occlusal surface of the crown should preferably retain a mild wear condition over time for increased resistance to sliding contact fracture [45].

Another important finding of this study is the absence of contact-induced cracks in the surface and sub-surface of the wear scars in zirconia. The material may be worn by enamel resulting in an aggressive wear. Obviously, the material wear characteristics are best determined through clinical trials in spite of their expensiveness and time-consuming nature. This limits the preliminary testing of the potential restorative in vitro material evaluation, though the wear simulation can show the trend for clinical application.

The COF mean value observed for the specimens in the five groups showed significant differences for these specimens. The overall conditions imposed in the study were harsher than the ones observed in the oral cavity. It is expected that the restorative material should possess wear properties similar to that of natural teeth, which is essential for the reduction of induced pathological consequences. The wear behaviour of polished zirconia showed the least wear rate among all the groups. The typical evolution of the COF during sliding for all the tested specimens is shown in Figure 10 (COF). The running-in period revealed a significant increase in the friction values. The COF values of all the specimens stabilized after 1 h of sliding. The steady-state COF values were quantified based on the reciprocating sliding of the dental restorative material against the zirconia ball in the presence of artificial saliva. The sliding scratches observed were attributed to the abrasive wear.

Various attempts to obtain the 3-D measurements of surface asperities have been reported in the literature. These studies have limitations such as the stylus tip radius, difficulties in positioning, and identification of subtle measuring points. These complications can be overcome by 3-D measurement as the surface topography is three- dimensional in nature [46,47]. Hence, Sa measurements were used in the present work. The surface roughness of the material decreased after wear. The 3-D surface parameters, namely Vvv and Svk, gave a better insight into the suitability of the groove depth for oral cavity retainment. The G5 values are minimal as shallow grooves were produced after wear.

It is shown that a controlled laboratory is able to identify and measure wear susceptibility under conditions that are representative of basic occlusal contact. The results also indicate that wear damage, in addition to producing substantial material loss, can result in an early tooth or prosthesis failure.

As emphasised in the introduction, the present study focuses on the dental adjustment procedures (sandblasting, polishing process) and their influence on the mechanical properties (surface roughness, hardness, SEM, XRD, and COF) of the Y-TZP specimens. Based on the results in the preceding section, the hypothesis that post-fabrication processes have significant influence on the surface of the material was accepted.

## 5. Conclusions

The impact of occlusal contact on wear is important. Averaged 2-D height characteristics were used to investigate the surface-produced wear due to its broad use and popularity. Surface metrology’s full potential for industrial process control is limited because it can only supply limited information. Many 3-D factors must be investigated under in vitro settings to determine the material’s lifetime. In this regard, the 3-D properties of Y-TZP specimens treated to various post-fabrication procedures were determined. The impacts of post-processing activities such as polishing and sandblasting on the surface topography, as well as related quantitative research, were also explored. Because both the polish and the sandblasting surface are crucial to the system’s performance, they should be treated with care to avoid restorative material damage.

## Figures and Tables

**Table 1:** Surface roughness values of the specimens subjected to different surface treatments before wear and after wear.

Post-Fabrication Processes	3-D Surface Roughness before Wear			
	Average Roughness Sa (µm)	Root Mean Square Roughness Sq (µm)	Dale Void Volume Vvv (µm³/µm²)	Reduced Valley Depth Svk (µm)
G1:S (S.D)	1.168 (0.495)	1.431 (0.579)	0.144 (0.05)	1.189 (0.405)
G2:S + P (S.D)	0.687 (0.091)	0.878 (0.124)	0.125 (0.026)	1.192 (0.261)
G3:S + P + S.B (S.D)	1.377 (0.362)	1.726 (0.425)	0.213 (0.029)	1.882 (0.203)
G4:S + S.B (S.D)	1.189 (0.302)	1.481 (0.380)	0.155 (0.052)	1.283 (0.56)
G5:S + S.B + P (S.D)	0.927 (0.307)	1.162 (0.35)	0.175 (0.027)	1.489 (0.218)
**Post-Fabrication Processes**	**3-D Surface Roughness after Wear**			
	**Average Roughness** **Sa (µm)**	**Root mean Square Roughness** **Sq (µm)**	**Dale Void Volume Vvv (µm³/µm²)**	**Reduced Valley Depth Svk (µm)**
G1:S (S.D)	0.723 (0.104)	0.957 (0.139)	0.17 (0.033)	1.689 (0.332)
G2:S + P (S.D)	0.507 (0.034)	0.660 (0.036)	0.116 (0.01)	1.148 (0.069)
G3:S + P + S.B (S.D)	1.049 (0.105)	1.354 (0.106)	0.189 (0.009)	1.832 (0.124)
G4:S + S.B (S.D)	0.794 (0.052)	1.029 (0.060)	0.175 (0.008)	1.752 (0.077)
G5:S + S.B + P (S.D)	0.182 (0.018)	0.227 (0.021)	0.032 (0.003)	0.312 (0.035)

**Table 2:** Results of the one-way ANOVA for Sa with respect to the different fabrication processes.

Source	DF	Adj SS	Adj MS	F-Value	*p*-Value
Different fabrication process	4	1.337	0.3342	2.40	0.084
Error	20	2.780	0.1390		
Total	24	4.116			
